# Plasma metabolomic response to high-carbohydrate meals of differing glycaemic load in overweight women

**DOI:** 10.1007/s00394-023-03151-7

**Published:** 2023-04-21

**Authors:** Brenan Durainayagam, Cameron J. Mitchell, Amber M. Milan, Marlena C. Kruger, Nicole C. Roy, Karl Fraser, David Cameron-Smith

**Affiliations:** 1 Liggins Institute, University of Auckland, Auckland, New Zealand; 2 Department of Epidemiology and Biostatistics, School of Public Health, Imperial College London, Norfolk Place, London, UK; 3 School of Kinesiology, The University of British Columbia, Vancouver, BC Canada; 4 Food & Bio-Based Products Group, AgResearch, Palmerston North, New Zealand; 5 High-Value Nutrition, National Science Challenge, Auckland, New Zealand; 6 School of Health Sciences, College of Health, Massey University, Palmerston North, New Zealand; 7 The Riddet Institute, Massey University, Palmerston North, New Zealand; 8 Department of Human Nutrition, University of Otago, Dunedin, New Zealand; 9 Colleges of Health, Medicine and Wellbeing, and Engineering, Science and Environment, The University of Newcastle, University Drive, Callaghan, NSW 2308 Australia

**Keywords:** Metabolomics, Metabolic syndrome, Protein, Postprandial

## Abstract

**Background:**

Metabolomic dysregulation following a meal in overweight individuals with the Metabolic Syndrome (MetS) involves multiple pathways of nutrient storage and oxidation.

**Objective:**

The aim of the current study was to perform an acute cross-over intervention to examine the interactive actions of meal glycaemic load (GL) on the dynamic responses of the plasma metabolome in overweight females.

**Methods:**

Postmenopausal women [63 ± 1.23y; Healthy (*n* = 20) and MetS (*n* = 20)] ingested two differing high-carbohydrate test meals (73 g carbohydrate; 51% energy) composed of either low glycemic index (LGI) or high (HGI) foods in a randomised sequence. Plasma metabolome was analysed using liquid chromatography–mass spectrometry (LC–MS).

**Results:**

In the overweight women with MetS, there were suppressed postprandial responses for several amino acids (AAs), including phenylalanine, leucine, valine, and tryptophan, *p* < *0.05*), irrespective of the meal type. Meal GL exerted a limited impact on the overall metabolomic response, although the postprandial levels of alanine were higher with the low GL meal and uric acid was greater following the high GL meal (*p* < *0.05*).

**Conclusions:**

MetS participants exhibited reduced differences in the concentrations of a small set of AAs and a limited group of metabolites implicated in energy metabolism following the meals. However, the manipulation of meal GL had minimal impact on the postprandial metabolome. This study suggests that the GL of a meal is not a major determinant of postprandial response, with a greater impact exerted by the metabolic health of the individual.

*Trial registration* Australia New Zealand Clinical Trials Registry: ACTRN12615001108505 (21/10/2015)

**Supplementary Information:**

The online version contains supplementary material available at 10.1007/s00394-023-03151-7.

## Introduction

The metabolic syndrome (MetS) is a cluster of conditions highly predictive for the subsequent development of type 2 diabetes (T2D) and cardiovascular disease (CVD) [1, 2]. MetS is characterised by the International Diabetes Federation (IDF) as central adiposity, plus two of either; elevated blood pressure, dyslipidaemia [low high-density lipoprotein (HDL), high low-density lipoprotein (LDL), and triglycerides] and/or elevated fasting glucose [1]. Whilst there are established cut-off values for just a small number of circulating biochemical intermediates, there are complex alterations in the abundances of many hundreds of metabolites [3, 4]. Therefore, many metabolic pathways are impacted, contributing to heterogeneity of disease aetiologies that are predicted by MetS [5].

Analysis of the metabolomic complexity of MetS is most frequently conducted on the fasting state, although it is apparent that disordered metabolic flux is evident following nutrient ingestion [6, 7]. To gain further insight into the transient metabolomic responses to nutrient ingestion, a simplified strategy is to utilise an oral glucose tolerance test (OGTT). The OGTT when combined with the use of high-throughput metabolomic technologies has further reinforced the complexity of biochemical pathways affected by MetS, including amino acid metabolism [6–10]. Unlike an OGTT, meals are a highly variable mix of nutrients [7, 9, 11, 12]. For high-carbohydrate meals, the impact of the meal composition on postprandial glycaemic responses has been extensive investigated as a meal variable that can be manipulated to impact on longer term health risks [13]. Lower GI of individual foods, or when applied to the calculated sum of ingested carbohydrates in the overall meal, glycaemic load (GL), has been used as a dietary strategy to improve metabolic health [14]. Diverse metabolites indicative of adaptations in multiple metabolic pathways are impacted by dietary manipulation of meal GL [15]; however, whether differences in the GL of a single meal exert impact on the complex postprandial metabolome is not yet known.

Therefore, the aim of this study was to examine the complex metabolomic responses to mixed test meals, containing either high glycaemic index (HGI) or low GI (LGI) carbohydrates in a cross-over study. For this, a cohort of female participants selected on the basis of the presence or absence of MetS were recruited. Analysis was undertaken of metabolome profiling using hydrophilic interaction chromatography (HILIC) coupled with high-resolution mass spectrometry (HRMS). Based on the available literature, it was hypothesised that using an exploratory metabolomics approach, dynamic alterations in amino acids and related metabolites would be identified and these metabolites would be a key discretionary feature of the altered circulating metabolomic responses to carbohydrate-rich meals [9, 16, 17].

## Methods

### Ethics

Written informed consent was obtained from all subjects. The experimental protocol was reviewed and approved by the University of Auckland Human Participants and Ethics Committee (Ref #014501). The trial was retrospectively registered at Australia New Zealand Clinical Trials Registry (ANZCTR; ACTRN12615001108505).

### Participants

The study recruited 40 postmenopausal Caucasian women from the Auckland region through newspaper advertisements and from the university community. Eligible subjects were required to have a BMI between 18 and 34 kg/m^2^ and aged between 55 and 70 years. Individuals with a medical history of cardiovascular or metabolic disease/conditions, and who were currently taking medications that may interfere with study endpoints were excluded from further participation in the trial. The allocation ratio was 1:1 into two groups; MetS, assigned according to the IDF guidelines [1] or who did not have established risk factors and body mass index (BMI) was in the healthy range (18 to 25 kg/m^2^).

Due to the complexity of untargeted metabolomics, there remains no standard method for sample size estimation [18, 19]. Therefore, practically, we adopted a sample size based on previous research examining postprandial responses to test meal ingestion plus the economic, ethical, and logistical constraints of our study design and funding sources [20].

### Experimental design

The randomised cross-over trial was conducted at the Maurice and Agnes and Paykel Clinical Research Unit at the Liggins Institute, University of Auckland, Auckland, New Zealand. The two mixed meals (Table 1) were formulated to be equal in all macronutrients; carbohydrates (73 g), protein (40 g), fat (13 g), and energy (~ 2380 kJ), with the primary difference being the glycaemic index (GI) of the included carbohydrates. Each food item was matched directly to those in the University of Sydney GI Online Database [21]. Foods that were not available in the database were matched to the available foods listed with similar characteristics, to achieve estimates of GI value. The recommended formula for the calculation of daily GL was based on the previous studies [22] and is as follows:

$$\sum \limits_{i = 1}^{n} {\text{GI}}\;{\text{of}}\;{\text{each}}\;{\text{food}}\;{\text{item}} \times {\text{CHO}}\;{\text{content}}\;{\text{in}}\;{\text{food}}.$$

**Table 1:** Macronutrient composition of each meal

	Serving size	Calories (kcal)	Protein (g)	Fat (g)	Carbohydrates (g)	GI	GL
Low glycaemic							
WPI protein (g) unflavoured	30	116	27.7	0.3	0.15	0	0
Anchor butter (g)	10	72	< 1.0	8.1	< 1.0	0	0
Grains bread (slices)	3	297	12.6	4.7	48.5	41	27
Fructose (g)	25	100	0	0	25	0	3
*Total*		585	40.1	13.1	73.6	51	30
Macronutrient composition (%)			28.1	20.1	51.6		
High glycaemic							
WPI protein unflavoured (g)	30	116	27.7	0.3	0.15	0	0
Anchor butter (g)	12.75	92.3	< 1.0	10.4	< 1.0	0	21
White bread (slices)	4	272	11.2	2.4	46.6	70	0
Maltodextrin (g)	9	36	0	0	9	110	44
Gatorade orange (ml)	300	73.2	0	0	18	89	13
*Total*		589	38.9	13.1	73.6	269	79
Macronutrient composition (%)			27.1	20.0	51.3		

*WPI* whey protein isolate, *GI* glycaemic index, *GL* glycaemic load

Participants were advised to maintain their dietary habits, body weight, and physical activity levels, with strenuous exercise, dietary supplements, and alcohol consumption suspended 2 days before trial days. Subjects arrived fasted (overnight) where anthropometric data were collected before a catheter was inserted into an antecubital vein and a baseline sample (time 0) was taken followed by consumption of the breakfast within 10 min. Four postprandial samples were collected for metabolomics analysis (30, 60, 120, and 300 min) into EDTA blood collection tubes (BD, Mt Wellington, New Zealand). Samples were centrifuged at 1500×*g* for 15 min at 4 °C, and the supernatants collected in microtubes and stored at − 80 °C until analysis.

### Metabolomic analysis

The extraction was performed using a slightly modified protocol based on the method used by [23]. The modifications to the protocol included a reduced volume of plasma due to oversaturation observed on the analytical instruments used at higher volumes. In addition, a 1:1 (v/v) chlorform:methanol mixture deviated from the Folch protocol which is a 2:1 (v/v) chloroform:methanol composition. Briefly, 100 µl were extracted by liquid–liquid extraction using a chilled (− 20 °C) mixture of 800 µl chloroform:methanol (1:1), followed by agitation and held at − 20 °C for 30 min. 400 µl of water was then added, the sample vortexed for 30 s and centrifuged for 15 min at 12,500×*g* at room temperature to separate the aqueous (upper) and organic (lower) phases. 250 µl of the aqueous phase was evaporated to dryness under a stream of nitrogen and reconstituted in 300 µl of acetonitrile:water (1:1) containing 0.1% formic acid and 10 µg/ml d_2_-tyrosine as an internal standard. Blank samples were prepared exactly as the test samples, but plasma was replaced with Milli-Q water. Samples were randomised before extraction to avoid systematic analytical batch and run-order effects. To verify and maintain data quality, a quality control (QC) sample, comprising a pooled extract of all samples, was injected once every ten samples. Retention time, signal/intensity, and mass error of internal standards were monitored to check instrument response variability and retention time shifts. Normalisation of metabolites was obtained in both positive and negative ionisation modes, and thus, there was no run-order impact on the analysis (Figure S1).

### Liquid chromatography–mass spectrometry

Plasma extracts and blanks were analysed through LC–MS streams using both positive and negative ionisation modes separately, as previously described [24]. Briefly, the Thermo LC–MS system (Thermo, Waltham, MA, USA) consisted of Acela 1250 quaternary UHPLC pump, a PAL auto-sampler fitted with a 15,000 psi injection loop. A Merck polymeric bead-based ZIC-pHILIC column (100 mm × 2.1 mm, 5 µm; Merck, Darmstadt, Germany) was used for chromatographic separation. The column was connected to an Exactive Orbitrap mass spectrometer with electrospray ionisation (Thermo, San Jose, CA). The mobile phase was a combination of acetonitrile-formic acid (99.9:0.1, v/v; solvent A) and water–ammonium formate (16 mM, pH 6.3; solvent B). The samples were separated at 25 °C with a flow rate of 250 µl/min and a gradient elusion programme as follows: 97% A (0–1 min), 97–70% A (1–12 min), 70–10% A (12–14.5 min), held at 10% A (14.5–17 min), and then returned to 97% A (17–18.5 min). Finally, the gradient was held for further 5.5 min to equilibrate prior to the next injection. Samples were run in both positive and negative ionisation modes separately. The positive ionisation parameters were as follows: spray voltage, 3.5 kV; capillary temperature, 325 °C; capillary voltage, 90 V, tube lens 120 V. Negative ionisation parameters were as follows; spray voltage, − 3.0 kV; capillary temperature, 325 °C; capillary voltage − 90 V, tube lens − 100 V. The nitrogen source gas desolvation settings were the same both modes (arbitrary units); sheath gas 40; auxiliary gas 10; sweep gas 5. Mass spectral data were collected in profile data acquisition mode covering a mass range of *m/z* = 55–1100 with mass resolution setting of 25,000 and a maximum trap fill time of 250 ms using the Xcalibur software package (Thermo, San Jose, CA).

### Data integration

XCMS software [25] was used for peak detection, alignment, and noise elimination. The resultant peak table generated was subjected to run-order correction and batch normalisation utilising pooled QC samples and applying the LOESS regression model [26]. Features with a CV of > 30% within the pooled QC samples were excluded.

### Statistical analysis

Metabolomics data were analysed with a linear mixed-effects model (LMM) approach. Statistical analysis was performed using R (version 3.1.2) [27]. The “nlme” package was used to perform LMM [28]. Fixed effects/predictors for the LMM were assigned as; ‘meal’ which is the difference between the LGI and HGI meal; ‘status’ which represents the difference between controls and MetS; ‘time which denotes differences across the five time points’ and the random effect being ‘participant’. For LMM, *p *values of overall effects was determined using conditional *F* tests with Kenward–Roger correction degrees of freedom as implemented in the ANOVA function from the package car (version 2.0-21). *P* values for differences between levels of categorical predictors were determined using parametric bootstrapping as implemented in lme permmodels function, with 1000 permutations. There are three levels of results produced by LMM: the significance of the overall model for each metabolite, the significant of the independent variables and their interactions at the status, meal, time, meal-by-time, status-by-time, meal-by-status-by time (three-way interaction) and (for post hoc testing) the significance of between-groups and within-groups effects at each time point. Multiple comparisons were corrected by controlling the false discovery rate (FDR; *p* < *0.05*) [29], using the R “predictmeans” package (version 1.0.1) [30]. The tidyverse package in R was used for graphics and additional analyses [31].

### Compound identification

Annotation was performed on significant features generated from LMMs for each interaction by matching peak identification data (accurate mass and retention time) against a local library of authentic standards run under identical conditions. If no hit was obtained, significant features were searched against the public domain databases HMDB and METLIN [32]. Mass error tolerance of 5 ppm was used. Feature annotation was based on the level of confidence according to The Metabolomics Standards Initiative [33] (Table S1).

## Results

**Fig. 1:**
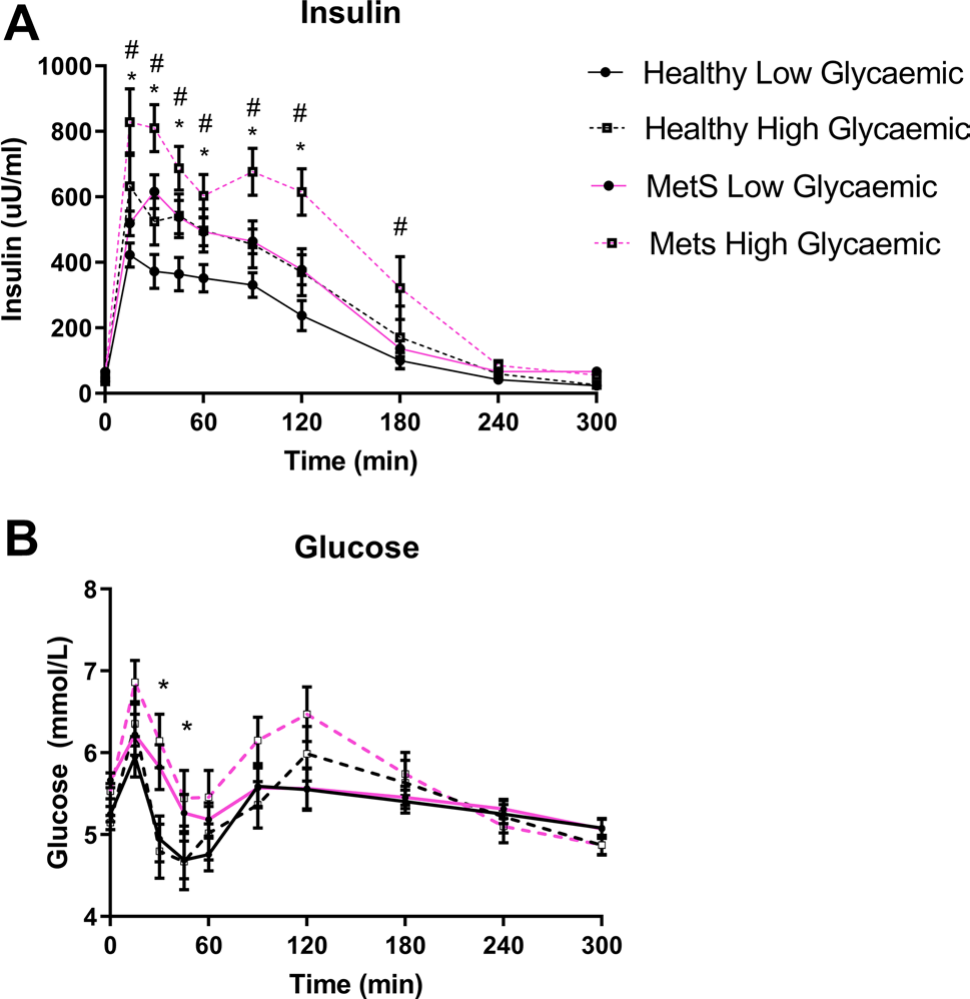
**A** Insulin and **B** glucose postprandial response. *p < *0.05* between healthy and MetS. ^#^*p* < *0.05* between LGI and HGI. Error bars represent standard error of mean

The women recruited into the study on the basis of the MetS classification had larger waist circumference (*p* < *0.001*), higher BMI (*p* < *0.001*), elevated fasting plasma glucose (*p* = *0.003*), and triglycerides (*p* < *0.01*), compared to the healthy women. They also had reduced plasma HDL (*p* = *0.027*) compared to the women within the healthy weight range (Table 2). In response to the meals, the MetS women exhibited greater postprandial insulin concentrations (15 min to 120 min, post-meal; (Fig. 1a; *p* < *0.05)* after both the HGI and LGI meals. There was also a status × time interaction (*p* = *0.011*) for postprandial glucose response, with MetS women displaying greater plasma glucose concentrations at 30 and 45 min postprandial, compared to the healthy women (Fig. 1b; *p* < *0.05*).

**Table 2:** Baseline clinical characteristics

	Healthy adults (*n* = 20)	MetS adults (*n* = 20)
Subject characteristics		
Age (years)	63.4 ± 1.00	63.00 ± 1.26
BMI (kg/m^3^)	24.3 ± 0.70	29.00 ± 0.66***
ALT	12.59 ± 1.71	21.81 ± 2.78*
HOMA-IR	1.48 ± 0.15	3.00 ± 0.27***
IDF measurements for MetS		
Waist circumference (cm)	80.4 ± 2.2	93.8 ± 1.7***
Systolic blood pressure (mmHg)	121.6 ± 3.0	139.2 ± 3.0***
Diastolic blood pressure (mmHg)	67.0 ± 2.2	72.0 ± 2.3
Triglycerides (mM)	0.91 ± 0.06	1.40 ± 0.10*
HDL (mM)	2.1 ± 0.08	1.7 ± 0.1**
Fasting plasma glucose (mmol/l)	5.51 ± 0.12	6.0 ± 0.11*
Clinical characteristics		
% Body fat total	36.25 ± 2.10	45.01 ± 1.80
Creatinine (mmol/l)	74.17 ± 3.73	56.38 ± 6.48
AST (mmol/l)	43.19 ± 17.1	32.6 ± 6.71

Values represent means ± SEM. Amino acid values measured in µmol/l

*HOMA-IR* homeostatic model assessment of insulin resistance

Significance was determined by Student’s *t* test. *p < 0.05, **p < 0.01, ***p < 0.001 compared with MetS adults

**Fig. 2:**
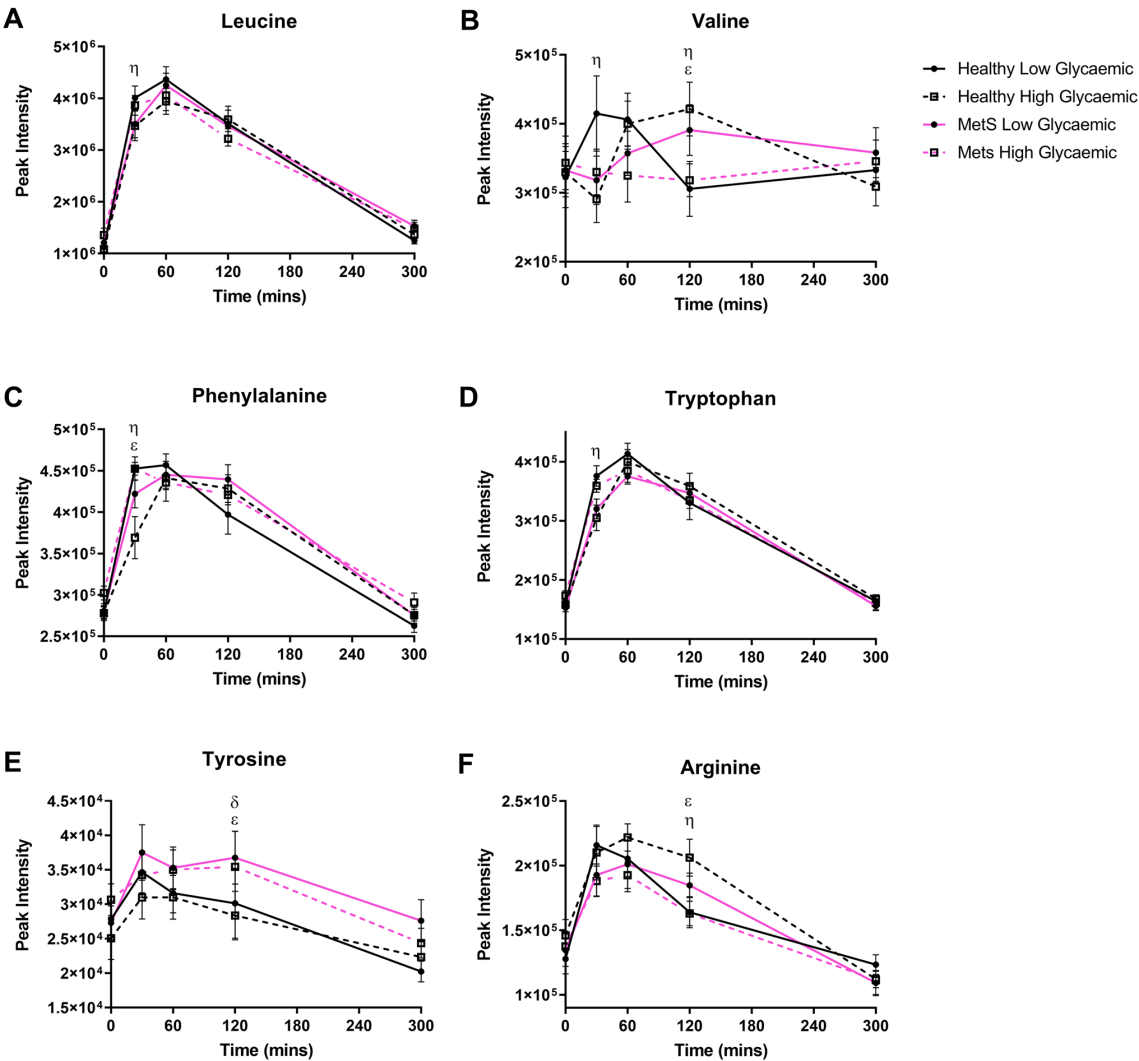
Postprandial amino acid which exhibited a three-way interaction. Values represent mean ± SEM in peak intensity. ^η^*p* < 0.05 represents difference between LGI and HGI in healthy women, ^λ^*p* < 0.05 represents difference between LGI and HGI in MetS women, ^δ^*p* < 0.05 difference between healthy and MetS women for HGI, ^ε^*p* < 0.05 difference between healthy and MetS following consumption of LGI

A total of 203 metabolite features were extracted, with 140 and 63 detected in positive and negative ionisation modes, respectively. In the postprandial phase, 23 metabolites exhibited significant interactions after LMM analysis, with either three-way interactions (status × meal × time) or two-way interactions (either status × time or meal × time). These interactions were predominately AA species. Figure 2 includes those features that were distinguished on the basis of a statistically significant three-way interaction. At 30 min, the LGI meal was higher than the HGI meal only in healthy women for leucine and valine, respectively (Fig. 2a, b; *p* < *0.05*). In addition, valine was higher at 60 min in the HGI meal than the LGI meal only in healthy women (Fig. 2b;* p* = *0.02*)*.* Phenylalanine and tryptophan at 30 min were higher in the LGI meal compared to the HGI meal only in healthy women (Fig. 2c, d; *p* < *0.05*). In addition, the healthy women had a higher abundance of phenylalanine at 30 min compared to the MetS women only when the LGI meal was consumed (*p* = *0.005*)*.* Tyrosine demonstrated two three-way interactions; the first being a difference only in the LGI meal with an increased peak in MetS women compared to healthy women, at 120 min. The second difference was following the HGI meal at 120 min, being higher in MetS women compared to healthy women (Fig. 2e; *p* = *0.038*)*.* Arginine was higher in the HGI meal compared to the LGI meal at 120 min only in healthy women. Also for arginine at 120 min, in the HGI meal only, it was higher in healthy women compared to MetS women (Fig. 2f; *p* < *0.05*).

**Fig. 3:**
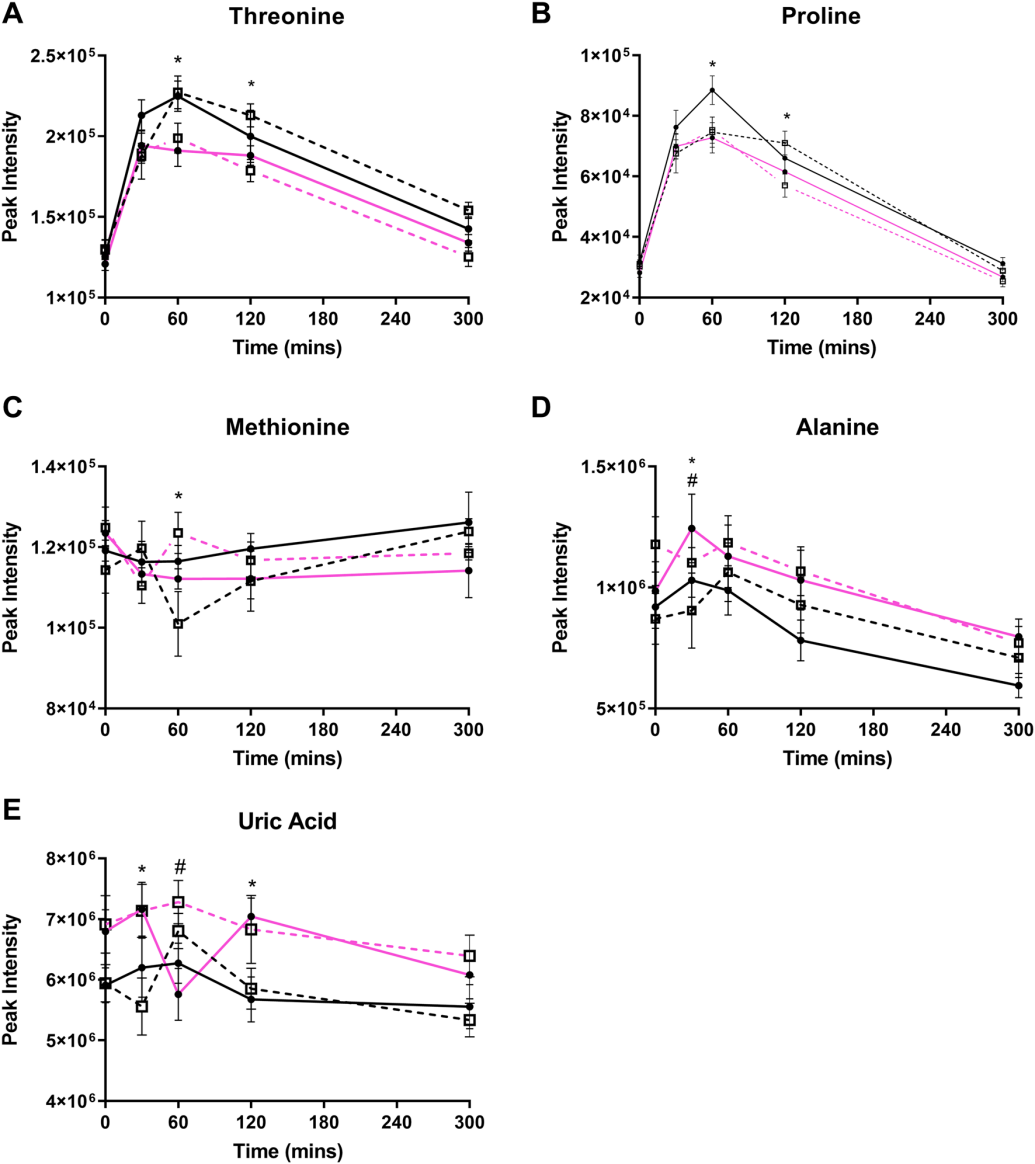
Postprandial significant metabolites differentiated by a two-way interaction. Values represent mean ± SEM in peak intensity. * Denotes a significant difference between MetS and healthy women. # Represents a significant difference between HGI and LGI

Figure 3 shows the AA’s that demonstrated a two-way interaction. Threonine and proline were reduced in the MetS women, compared to healthy women at 60 min and 120 min (Fig. 3a, b; *p* < *0.05*). At 30 min, alanine and uric acid were higher in MetS women compared to healthy women irrespective of meal (Fig. 3d, e; *p* < *0.05*).

**Fig. 4:**
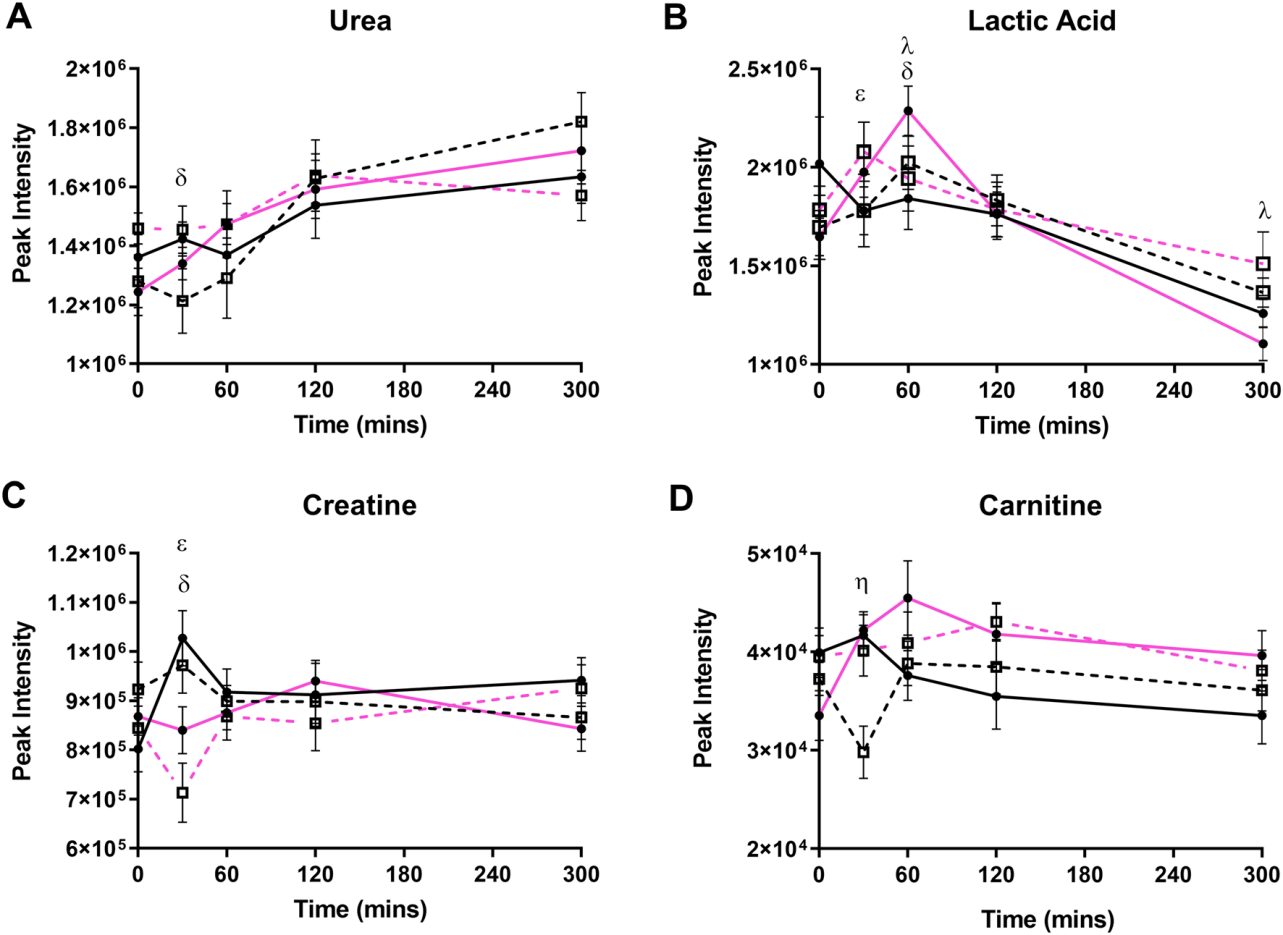
Non-amino acids that exhibited a three-way interaction. Values represent mean ± SEM in peak intensity. ^η^*p* < 0.05 represents difference between LGI and HGI in healthy women, ^λ^*p* < 0.05 represents difference between LGI and HGI in MetS women, ^δ^*p* < 0.05 difference between healthy and MetS women for HGI, ^ε^*p* < 0.05 difference between healthy and MetS following consumption of LGI

Metabolites other than amino acids that were also identified as exhibiting a three-way interaction are shown in Fig. 4. Urea was higher in MetS women compared to healthy women only post-HGI meal at 30 min (Fig. 4a; *p* = *0.015*)*.* At 30 min, lactic acid was higher in MetS women, compared to healthy women, in the HGI meal only (Fig. 4b; *p* = *0.028*). However, at 60 min, lactic acid was higher in MetS women compared to the healthy women post-LGI intake only (Fig. 4b; *p* = *0.020*)*.* Creatine demonstrated two three-way interactions, with the first being in the LGI intake only, where healthy women had a higher abundance compared to MetS women (Fig. 4c; *p* = *0.02*). The second interaction was only seen in the HGI meal only, where healthy women had a higher amount of creatine compared to MetS women (Fig. 4c; *p* = *0.005*). Carnitine reduced after the LGI compared to the HGI at 30 min only in healthy women (Fig. 4d; *p* = *0.01*). The remaining features that exhibited differences from the LMM are shown in Figure S2.

## Discussion

The current study compares the plasma metabolomic responses in postmenopausal women with or without MetS to two carbohydrate-rich meals, differing on the basis of GL. Using an untargeted LC–MS strategy, the results demonstrate a predominance of differences in circulating AAs between the healthy and MetS women. There were far fewer and more subtle differences in the circulating AA response between the HGL and LGL meals. Of the responses evident, these tended to be within the first hour following the meal, with no effect evident over the subsequent 2 h of analysis. The untargeted metabolomic analysis also identified metabolites related to energy utilisation pathways, including lactic acid and carnitine that exhibited a greater post-meal excursion in the women with MetS.

Despite the design of the meals to alter the calculated GL, the measured blood glucose concentrations demonstrated only a small transient heightened response in the MetS women, following ingestion of the HGL meal. For all study participants, blood glucose exhibited a bi-phasic response, with a nadir between 45 and 60 min after meal ingestion. Although the bi-phasic nature of blood glucose response to a mixed meal has been previously reported [34], the rapid onset and extent of the blood glucose nadir was unexpected. In following a large cohort using continuous glucose, monitoring the nadir in blood glucose is typically reported 2–3 h after a meal and may correspond with the onset of hunger [35].

Increased circulating BCAA has been demonstrated to be predictive of diabetes risk [36]. The current study and previous analysis demonstrated that there is no marked change in the plasma BCAA response to a mixed high-carbohydrate meal in individuals with MetS [16]. The measured differences in leucine occurred within 30 min of the meal and relative to the change in leucine from baseline was very small. It is unlikely that this subtle difference in leucine is of biological relevance. Plasma valine abundances fluctuated following all meals, with no evidence of a post-meal increase.

The dispensable amino acid tyrosine exhibited the tendency for increased abundances from 60 min till the study completion (300 min) in the MetS women, irrespective of the meal. These results are consistent with the observation of greater plasma tyrosine responses following different high protein meals in those individuals exhibiting insulin resistance [16]. This speculatively corroborates with the previous studies that have demonstrated a reduced clearance can augment the biosynthesis of norepinephrine and dopamine, both of which have been linked to glucose homeostasis [37, 38]. Arginine exhibited an increase in healthy women following the HGI meal, with MetS women demonstrating reduced levels. Arginine is critical in the activation and regulation of both AMPK and mTOR [39] and is central for nitric oxide synthesis, suggesting a role in energy regulation and vascular homeostasis [40, 41]. Further analysis is required to determine the extent to which these observed changes in tyrosine impact on downstream metabolites and function.

In the current study, further two-way associations in the response of several amino acids and energy metabolites was demonstrated. Of the identified AAs, alanine was notable in that the post-meal response tended to be greater in the women with MetS. Alanine provides a physiological balance for glucose and proline via glutamate in the tricarboxylic acid cycle (TCA) [42]. Alanine aminotransferase (ALT), is responsible for conversion of pyruvate to alanine, which was increased in MetS women in this trial and consistent with the previous observations suggesting liver dysfunction [43]. Concomitantly, alanine was elevated in MetS women in the postprandial phase for both meals, suggesting that its kinetics are largely influenced by metabolic risk. Conversely, plasma threonine and proline tended to be reduced in the MetS group, irrespective of the meal.

Of particular interest is whether variations in the GL of a meal can be identified to modify the concentration of metabolites indicative of altered oxidative metabolism. Lactic acid is the end product of glycolysis, with plasma concentrations indicative of flux in the Cori cycle, where it is exported from glycolytically active tissues into the plasma for synthesis to glucose in the liver [44]. Lactic acid concentrations have been reported as being elevated in insulin resistant individuals in the first 2 h following an OGTT, being speculated to be an indicator of the impaired oxidative capacity [45, 46]. In this study, there were subtle and transient impacts of both MetS and meal GL on the plasma concentrations of lactic acid up until 60 min. The small and transient nature of these changes suggests that in the context of the meal used in this study, the dynamic response of lactic acid is poorly indicative of MetS and did not differentiate between meals of high or low GI.

Fasted creatine has previously been associated with MetS [47] and plays a fundamental role in energy buffering. Creatine immediately increased by 15% in healthy women, whilst in contrast, it decreased by 10% in the MetS women. These differences, as with lactic acid, were evident only in the first postprandial sample (30 min) and did not subsequently persist. Such differences may suggest an acute difference in energy utilisation, but more careful analysis is required.

There are important considerations and limitations of the current study. These investigations are limited to older Caucasian females who are postmenopausal, and hence, caution is required in generalising these results to males, younger adults, or to multiple ethnicities. Furthermore, whilst metabolomics has provided significant insight into the differences between postprandial profiles of healthy and MetS women, it does not provide a detailed understanding of the variations in the either the rate of absorption or the rate of tissue clearances due to breakdown of meals between phenotypes. Additionally, the choice of meal is likely to impact on the extent to which these results can be generalised. Future studies can address this issue by incorporating stable isotopes into the food, enabling the precise analysis of metabolite fluxes. Given that there was no formal estimation of sample size calculation and the untargeted nature of the LC–MS analysis, this study should be viewed as a potential pilot to provide guidance for future investigations.

## Conclusion

In summary, the use of an untargeted metabolomic analysis of plasma samples in postprandial women demonstrated small and transient differences in a range of AAs and several energy-related metabolites for overweight women, characterised as having the MetS, when compared to aged-matched leaner and metabolic healthier women. Further, the choice of meals used in this study did not markedly impact on the measured metabolomic responses. This study therefore cannot convincingly demonstrate whether differences in meal GL are important in altering postprandial metabolism in a manner that can either be beneficial for body weight regulation and metabolic health. However, the data emphasise the complexity of the postprandial responses to meals, composed of whole food, with the continued need to develop a more detailed understanding of the systems’ biological responses to differing meal types.

## Supplementary Information

Below is the link to the electronic supplementary material.

Supplementary file 1 (DOCX 890 KB)

## Data Availability

The data that support the findings of this study are available from the corresponding author, [DCS], upon reasonable request.

## Abbreviations

AA: Amino acids
AAA: Aromatic amino acid
AUC: Area under the curve
BCAA: Branched chain amino acid
BMI: Body mass index
CVD: Cardiovascular disease
GI: Glycaemic index
IDF: International Diabetes Federation
LC–MS: Liquid chromatography–mass spectrometry
LMM: Linear mixed-effects model
MetS: Metabolic syndrome
OGTT: Oral glucose tolerance test
QC: Quality control
T2D: Type 2 diabetes
