# Supplementary material for: Plasma metabolomic response to high-carbohydrate meals of differing glycaemic load in overweight women
Source: Eur J Nutr. 2023 Apr 21;62(5):2257–67. doi: 10.1007/s00394-023-03151-7 (PMC10349757; doi:10.1007/s00394-023-03151-7)
Supplement: Supplementary file 1 — (DOCX 890 KB) [file 394_2023_3151_MOESM1_ESM.docx]

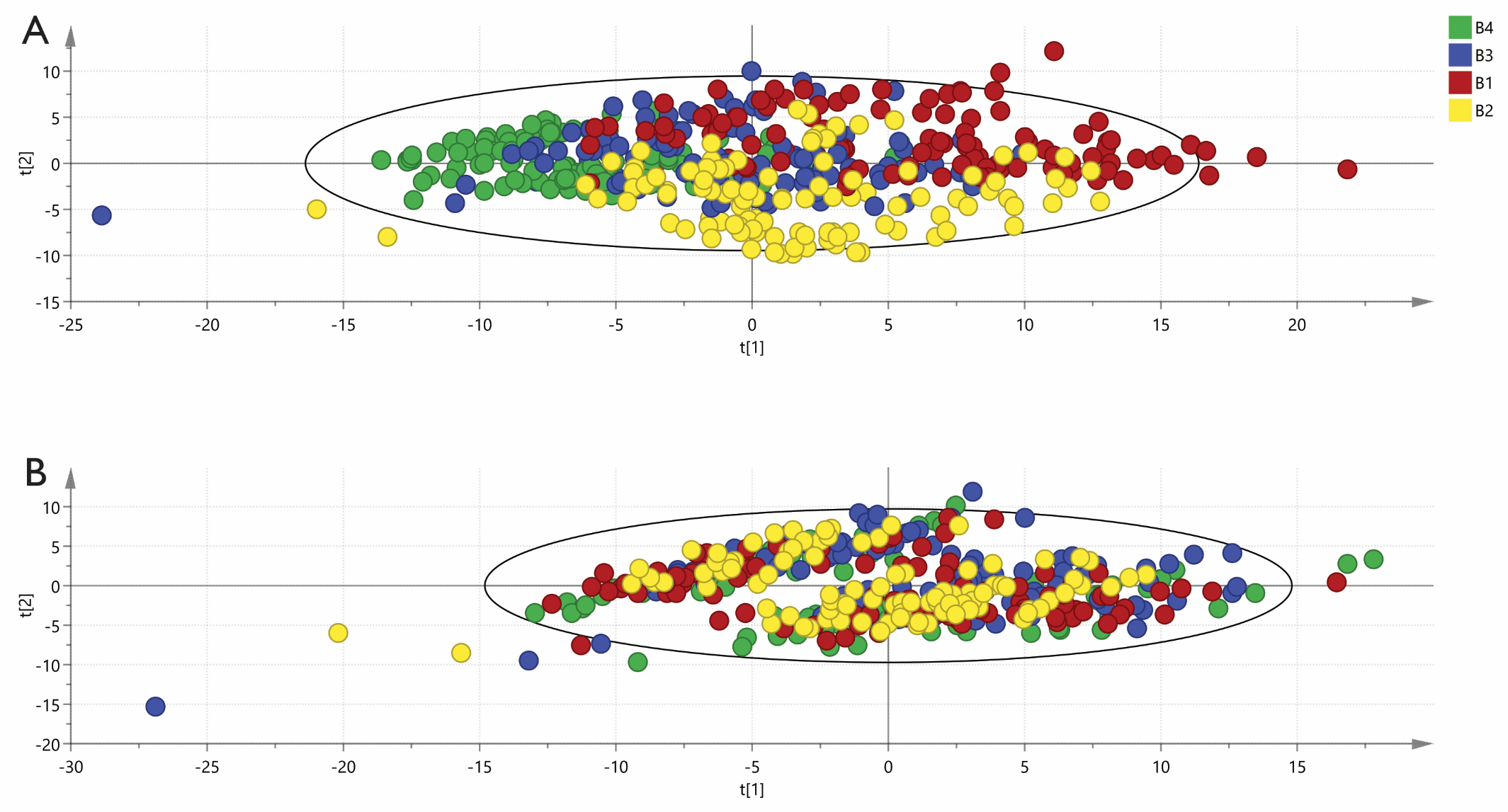


Figure S1: Batch order normalisation. (A) Representative PCA score plot before normalisation in WFM. (B) PCA score plot after batch normalisation using LOESS correction method in W4M.

Table S1: Class level identification

| Metabolite | m/z | Theoretical Mass | Ion | Rt (mins) | Ionisation mode | Class | Interaction | HMDB |
| --- | --- | --- | --- | --- | --- | --- | --- | --- |
| Urea | 61.0397 | 60.0553 | M+H | 7.51 | Positive | 1 | 3 | HMDB00294 |
| Tyrosine | 182.0812 | 181.1885 | M+H | 11.35 | Positive | 1 | 3 | HMDB0000158 |
| Valine | 119.0810 | 117.0790 | M+H | 9.51 | Positive | 1 | 3 | HMDB00883 |
| Phenylalanine | 166.0861 | 166.0863 | M+H | 10.11 | Positive | 1 | 3 | HMDB00159 |
| Leucine | 132.1022 | 132.1022 | M+H | 10.17 | Positive | 1 | 3 | HMDB00687 |
| Tryptophan | 205.0967 | 205.0969 | 2M+H | 10.69 | Positive | 1 | 3 | HMDB00929 |
| 5-Methoxytryptamine | 192.1001 | 190.2417 | M+H | 10.80 | Positive | 2 | 2S | HMDB0004095 |
| Carnitine | 162.0842 | 161.1989 | M+H | 10.94 | Positive | 2 | 3 | HMDB0000062 |
| Proline | 116.0708 | 116.0706 | M+H | 11.05 | Positive | 1 | 2M | HMDB00162 |
| Imidazole | 69.0449 | 68.0773 | M+H | 11.19 | Positive | 1 | 2M | HMDB01525 |
| Methionine | 150.0771 | 150.0586 | M+H | 11.39 | Positive | 1 | 2S | HMDB00696 |
| Creatine | 132.0764 | 132.0768 | M+H | 12.26 | Positive | 1 | 2S | HMDB00064 |
| Alanine | 90.0552 | 90.0558 | M+H | 12.42 | Positive | 1 | 2M | HMDB00161 |
| Dihydrothymine | 129.0656 | 128.0585 | M+H | 12.86 | Positive | 2 | 2M | HMDB00079 |
| Threonine | 120.0655 | 120.0655 | M+H | 12.89 | Positive | 1 | 2S | HMDB00167 |
| Arginine | 175.1194 | 175.1191 | M-H | 17.00 | Negative | 1 | 3 | HMDB00517 |
| Uridine | 243.0628 | 243.0617 | M-H | 9.22 | Negative | 1 | 2M | HMDB0000296 |
| Uric acid | 167.0208 | 168.1103 | M-H | 10.10 | Negative | 2 | 2M | HMDB0000289 |
| Lactic acid | 89.0248 | 89.0239 | M-H | 1.71 | Negative | 1 | 3 | HMDB0000190 |
| Unknown M141T616 | 141.1072 |  | M+H |  | Positive | 4 | 3 |  |
| Unknown M188T630 | 188.1281 |  | M+H |  | Positive | 4 | 3 |  |
| Unknown M302T793 | 171.0769 |  | M+H |  | Positive | 4 | 2S |  |
| Unknown M230T588 | 230.0961 |  |  |  | Negative | 4 | 3 |  |

Abbreviations; m/z, mass-to-charge ratio; Class 1: Identified marker confirmed by an authentic standard; Class 2: putatively identified marker based on elemental composition; Class 4: Unknown compound. Four levels of identification based on Metabolomics Standard Initiative

3: Three way interaction (status × meal × time)

2M: Meal × time interaction

2S: Status × time interaction


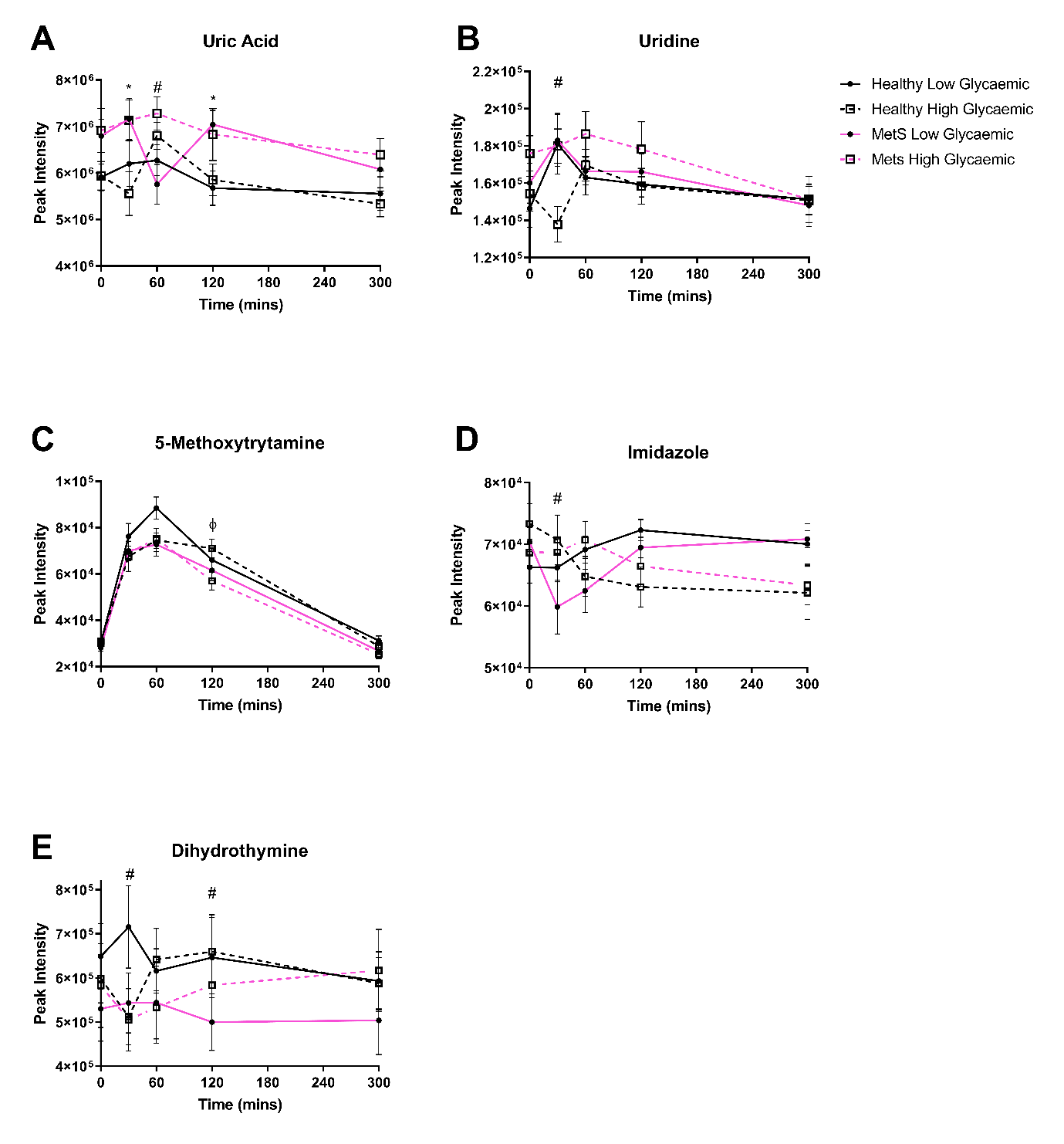


Figure S2: **Polar metabolites which exhibit two way interactions**. * *p< 0.05* between Healthy and MetS. # *p<0.05* between LGI and HGI. Error bars represent standard error of mean.
